# 2828. Incidence of Treatment Failure When Treated with Empiric Oral Antibiotics Among US Female Outpatients with Uncomplicated Urinary Tract Infection

**DOI:** 10.1093/ofid/ofad500.2439

**Published:** 2023-11-27

**Authors:** Debra L Fromer, Megan E Luck, Wendy Y Cheng, Malena Mahendran, Wilson L Da Costa, Megan Pinaire, Mei Sheng Duh, Madison T Preib, Jeffrey J Ellis

**Affiliations:** Hackensack University Medical Center / Hackensack Meridian School of Medicine, Hackensack, NJ; GSK, Collegeville, Pennsylvania; Analysis Group, Inc, Boston, Massachusetts; Analysis Group, Inc., Boston, Massachusetts; Analysis Group, Inc., Boston, Massachusetts; Analysis Group, Inc., Boston, Massachusetts; Analysis Group, Inc., Boston, Massachusetts; GSK, Collegeville, Pennsylvania; GSK, Collegeville, Pennsylvania

## Abstract

**Background:**

More than half of all women experience a urinary tract infection (UTI) in their lifetime, with most diagnoses classified as uncomplicated (uUTI). Management of uUTIs is impacted by treatment failure (TF), which may be driven by antibiotic (ABX) resistance and is associated with an increased risk of suboptimal outcomes. This study aimed to investigate the occurrence of TF to empirically prescribed oral ABX treatment in female outpatients with uUTI and characterize those failing versus not failing treatment.

**Methods:**

Retrospective data from Optum’s de-identified Clinformatics Data Mart Database dataset were used to assess female outpatients aged ≥12 years between January 2017 and September 2022 (**Figure 1**). Eligibility criteria included ≥1 uUTI diagnosis (i.e., no evidence of complicated UTI) with ≥1 empiric prescription of nitrofurantoin (NTF), trimethoprim-sulfamethoxazole (SXT), fluoroquinolones (FQ), fosfomycin, or beta-lactams (BL) within ±5 days of diagnosis (date of initial ABX prescription defined the index date), and ≥12 months of electronic health record activity before and after the index date. TF was defined as any of the occurring ≤28 days after the index date: second oral ABX prescription, administration of intravenous ABX, or a primary diagnosis of acute UTI in the emergency room or inpatient setting.

**Figure:**
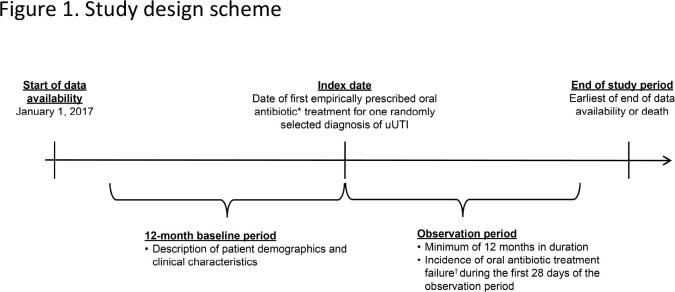

Data source: Optums de-identified Clinformatics Data Mart Database from January 1, 2017 to September 30, 2022. *Oral ABX treatments included NTF, SXT (separately, or in combination), FQ (i.e. levofloxacin, ciprofloxacin, and ofloxacin), fosfomyscin, and BL (i.e., amoxicillin, amoxicillin/potassium-clavulanate, ampicillin, cloxacillin, dicloxacillin, cefaclor, cefadroxil, cefdenir, cefditoren, cefditoren-pivoxil, cefixime, cefpodoxitil, cefprozil, ceftibuten, cefuroxime-axetil, cephalexin, cephradine, and loracarbef). †TF was defined as at least one of 1) a prescroption of a new oral ABX treatment for uUTI or second prescroption of the same empirically prescribed oral ABX treatment for uUTI within 28 days following the index date, 2) administration of an IV antibiotic treatment within 28 days following the index date, or 3) diagnosis of an acute UTI, defined as having a primary diagnosis of uncomplicated or complicated UTI in an acute care setting (i.e. inpatient or emergency department) within 28 daya following the index date. ABX< antibiotic; BL, beta-lactams; FQ, fluoroquinolone; IV, intravenous; NTF, nitrofrantoin; SXT, trimethoprim-sulfamethoxazole; TF, treatment failure; UTI, urinary tract infection; uUTI, uncomplicated urinary tract infection

**Results:**

Out of 376,004 empirically prescribed uUTI patients, 62,873 (16.7%) experienced TF, driven primarily by the need for a second oral ABX prescription (14.0%) (**Figure 2**; **Table 1**). On average, patients with TF were older than those without TF (48.9 vs 46.5 years, standardized difference [std diff]=12.3%) and had more pre-index prescriptions for oral ABX treatment (1.4 vs 0.9, std diff=23.5%). Notably, prior failure to oral ABX treatment (12.1% vs 4.7%, std diff=26.8%), recurrent UTI status (22.6% vs 17.0%, std diff=14.1%), and prior non-susceptibility to NTF, BL, or FQ (std diff=10.2–13.0%) were more frequent in patients with TF versus those not failing (**Table 2**).

**Figure 2.**
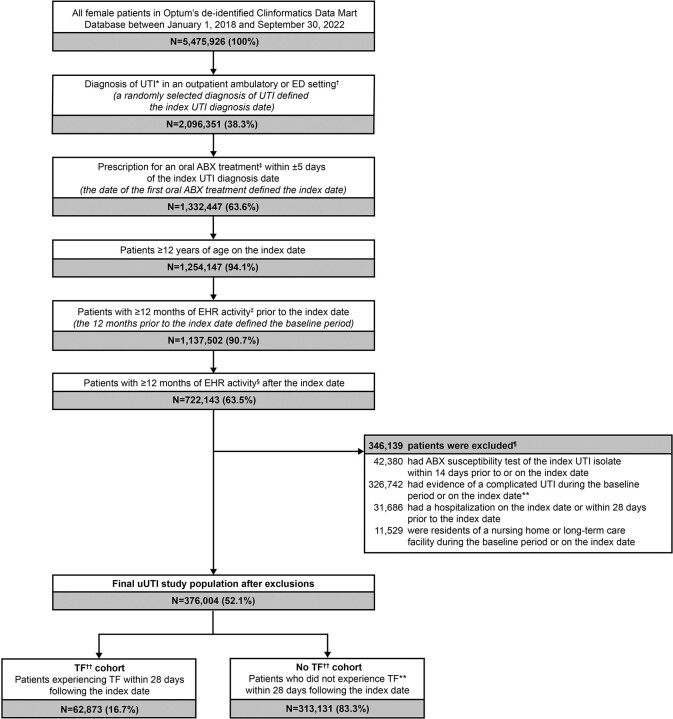
Patient selection flowchart. *Patients with a UTI diagnosis were identified using the following ICD-10-CM diagnosis codes: N30.0x, N30.9x, and N39.0. †Outpatient ambulatory settings included urgent care, ambulatory patient services, office or clinic, or telephone/online. ED settings included emergency patients only. ‡Oral ABX treatments included NTF, SXT (separately, or in combination), FQ (i.e., levofloxacin, ciprofloxacin, and ofloxacin), fosfomycin, and BL (i.e., amoxicillin, amoxicillin/potassium-clavulanate, ampicillin, cloxacillin, dicloxacillin, cefaclor, cefadroxil, cefdinir, cefditoren, cefditoren-pivoxil, cefixime, cefpodoxime-proxetil, cefprozil, ceftibuten, cefuroxime-axetil, cephalexin, cephradine, and loracarbef). Agents of each treatment class were identified using NDCs. §EHR activity was defined using the First Month Active (i.e., the earliest month and year with a recorded healthcare activity event) and Last Month Active (the latest month and year with a recorded healthcare activity event) fields in Optum’s de-identified Clinformatics Data Mart database. ¶Patients may have met the conditions of multiple exclusion criteria. Therefore, categories of exclusion criteria are not mutually exclusive and will not sum to the total number of patients excluded. **The following conditions were considered as evidence for a complicated UTI: 1) pregnancy, 2) diagnosed with urologic abnormalities, uncontrolled or complicated diabetes, or severe renal dysfunction, 3) immunosuppressed or treated with immunosuppressive therapy, 4) underwent relevant urological or nephrological procedures within 28 days prior to or on the index date or ureteral stent procedure during the baseline period, or 5) administered IV ABX within 28 days prior to or on the index date. Uncontrolled and complicated diabetes were identified using either ICD-10-CM codes or hemoglobin A1c>8. ††TF was defined as at least one of: 1) a prescription of a new oral ABX treatment for uUTI or second prescription of the same empirically prescribed oral ABX treatment for uUTI within 28 days following the index date, 2) administration of an IV ABX treatment within 28 days following the index date, or 3) diagnosis of an acute UTI, defined as having a primary diagnosis of uncomplicated or complicated UTI in an acute care setting (i.e., inpatient or ED) within 28 days following the index date. BL, beta-lactams; ED, emergency department; EHR, electronic health record; FQ, fluoroquinolones; ICD-10-CM, International Classification of Diseases, Tenth Edition, Clinical Modification; IV, intravenous; NDC, National Drug Code; NTF, nitrofurantoin; SXT, trimethoprim-sulfamethoxazole; UTI, urinary tract infection; uUTI, uncomplicated urinary tract infection.

**Figure:**
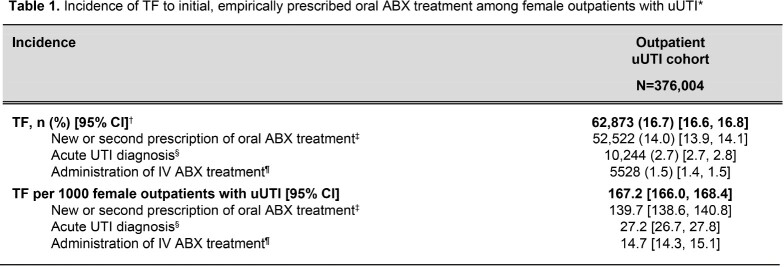

*Incidence of TF was evaluated over the first 28 days of the observation period following the index date (i.e., date of first empirically prescribed oral ABX treatment for one randomly selected diagnosis of uUTI). †Patients may have met multiple definitions of TF. Therefore, categories of TF are not mutually exclusive and will not sum to the total number of patients with TF. ‡Oral ABX treatments included NTF, SXT, FQ, fosfomycin, and BL. §Acute UTI was defined as having a primary diagnosis of uncomplicated or complicated UTI in an acute care setting (i.e., inpatient or emergency department). ¶IV ABX were identified using HCPCS codes. ABX, antibiotics; BL, beta-lactams; CI, confidence interval; FQ, fluoroquinolones; HCPCS, Healthcare Common Procedure Coding System; IV, intravenous; NTF, nitrofurantoin; SXT, trimethoprim-sulfamethoxazole; TF, treatment failure; UTI, urinary tract infecion; uUTI, uncomplicated urinary tract infection.

**Figure:**
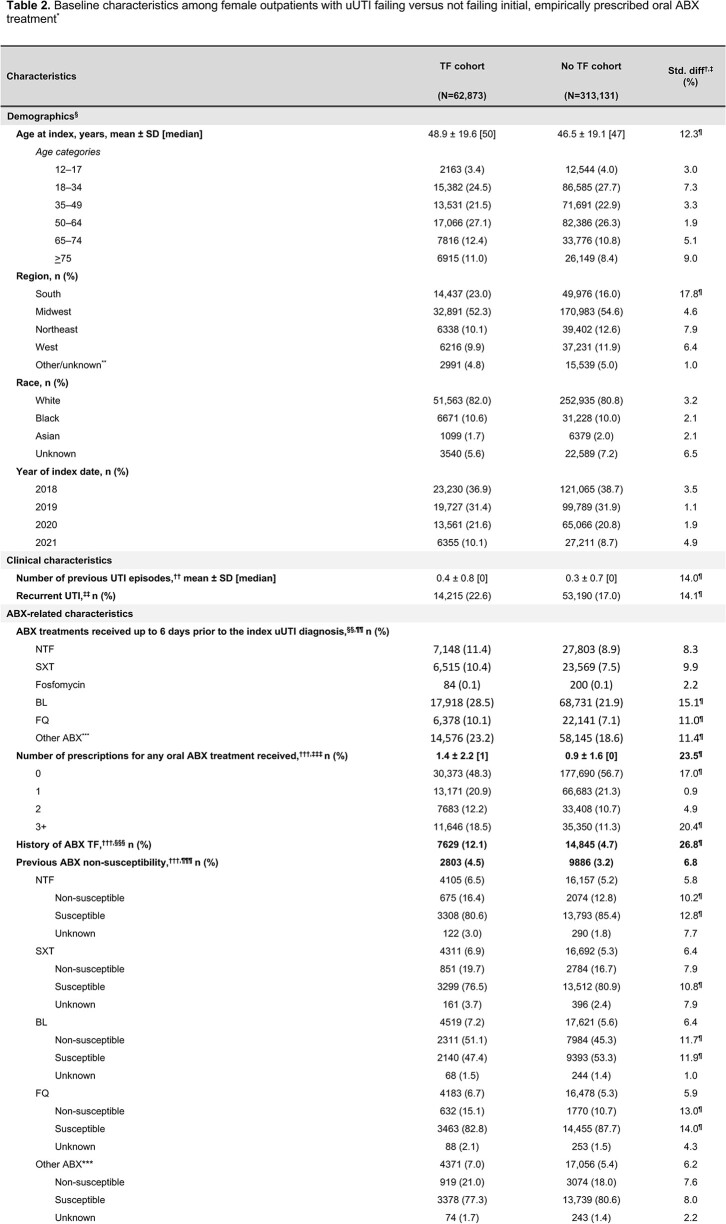

*TF was defined as at least one of 1) a prescription of a new oral ABX treatment for uUTI or second prescription of the same empirically prescribed oral ABX treatment for uUTI within 28 days following the index date, 2) administration of an IV ABX treatment within 28 days following the index date, or 3) diagnosis of an acute UTI, defined as having a primary diagnosis of uncomplicated or complicated UTI in an acute care setting (i.e., inpatient or ED) within 28 days following the index date. †For continuous variables, the std diff was calculated by dividing the absolute difference in means of the TF and no TF cohorts by the SD of both cohorts. The pooled SD was the square root of the average of the squared SD. ‡For dichotomous variables, the std diff was calculated using the following equation where P was the respective proportion of participants in each cohort: |(Pfailure-Pno failure)| / √[(Pfailure(1-Pfailure)+Pno failure(1-Pno failure))/2]. §Evaluated on the index date (i.e., date of first empirically prescribed oral ABX treatment for one randomly selected diagnosis of uUTI). ¶Std diff >10%, considered meaningful imbalance between cohorts. **As defined in the Optum EHR database, other/unknown region represents any region that did not fall under one of the four United States census bureau regions. ††The beginning of a UTI episode was defined using the date of the earliest diagnosis of UTI (identified through ICD-10-CM diagnosis codes and NLP in the Optum EHR database), during the baseline period up to and excluding the index uUTI diagnosis. Diagnoses occurring within 28 days of the initial diagnosis were considered part of the same initial episode. The beginning of subsequent episodes required a UTI diagnosis more than 28 days after the last diagnosis of the previous episode. ‡‡A patient was considered to have a recurrent UTI if 1) the patient was diagnosed with recurrent UTI (identified through NLP in the Optum EHR database) or 2) the patient had ≥3 UTI episodes during the 12-month baseline period, including the index UTI diagnosis, or ≥2 episodes in the 6 months prior to and including the index UTI diagnosis (diagnoses were identified using ICD-10-CM diagnosis codes). §§Evaluated from the 12 months up to the 3 days preceding the index uUTI diagnosis date. ¶¶ABX treatments were identified using NDCs and HCPCS codes. Any ABX treatments were considered, including but not limited to those used to treat uUTIs. ***Other ABX included any ABX agent that did not fall under the ABX classes of NTF, SXT, fosfomycin, BL, or FQ. †††Evaluated during the 12-month baseline period, not including the index date. ‡‡‡ABX prescriptions were identified using NDCs. §§§History of ABX TF was defined as evidence of TF for any previously treated UTI diagnoses occurring during the 12-month baseline period. TF was defined according to the definition listed in footnote†. ¶¶¶ABX non-susceptibility was identified through microbiology data in the Optum EHR database. A patient was considered non-susceptible if any of their isolates were tested for sensitivity to the ABX class of interest and the result was "Resistant" or "Intermediate." ESBL positivity was defined by either a non-susceptible test result (i.e., “Resistant” or “Intermediate”) to at least one third or fourth generation cephalosporin or a microorganism identified as an ESBL-producer (microbiology test performed on Enterobacterales isolates). ABX, antibiotic; BL, Beta-lactams; ED, emergency department; EHR, electronic health record; ESBL, extended-spectrum β-lactamase; FQ, Fluoroquinolones; HCPCS: Healthcare Common Procedure Coding System; ICD-10-CM, International Classification of Diseases, 10th Revision, Clinical Modification; IV, intravenous; NDC, National Drug Code; NLP, natural language processing; NTF, nitrofurantoin; SD, standard deviation; Std diff, standardized difference; SXT, trimethoprim-sulfamethoxazole; TF, treatment failure; UTI, urinary tract infection; uUTI, uncomplicated urinary tract infection.

**Conclusion:**

TF is considerable among empirically prescribed female outpatients with uUTI, occurring in nearly 17% of patients. Further investigation of observable demographics and clinical risk factors associated with TF may inform the unmet need for optimized ABX selection in the treatment of uUTI.

**Disclosures:**

**Debra L. Fromer, MD**, GSK: Advisor/Consultant|Johnson & Johnson/Janssen Pharmaceuticals: Advisor/Consultant **Megan E. Luck, PharmD**, GSK: Employee of, and shareholder in GSK **Wendy Y. Cheng, MPH, PhD, ORCID: 0000-0002-8281-2496**, Analysis Group, Inc.: Wendy Y. Cheng is an employee of Analysis Group, Inc., a consulting company that received funding from GSK to conduct this study **Malena Mahendran, MS**, Analysis Group, Inc.: Malena Mahendran is an employee of Analysis Group, Inc., a consulting company that received funding from GSK to conduct this study **Wilson L. Da Costa, MD, MPH, PhD**, Analysis Group, Inc.: Wilson L. da Costa is an employee of Analysis Group, Inc., a consulting company that received funding from GSK to conduct this study **Megan Pinaire, MPH**, Analysis Group, Inc.: Megan Pinaire is an employee of Analysis Group, Inc., a consulting company that received funding from GSK to conduct this study **Mei Sheng Duh, MPH, ScD**, Analysis Group, Inc.: Mei Sheng Duh is an employee of Analysis Group, Inc., a consulting company that received funding from GSK to conduct this study|ViiV Healthcare: Grant/Research Support **Madison T. Preib, MPH**, GSK: Employee|GSK: Stocks/Bonds **Jeffrey J. Ellis, PharmD, MS**, GSK: Jeffrey J. Ellis is an employee of, and shareholder in, GSK

